# Non-contiguous finished genome sequence of *Aminomonas paucivorans* type strain (GLU-3)

**DOI:** 10.4056/sigs.1253298

**Published:** 2010-11-20

**Authors:** Sam Pitluck, Montri Yasawong, Brittany Held, Alla Lapidus, Matt Nolan, Alex Copeland, Susan Lucas, Tijana Glavina Del Rio, Hope Tice, Jan-Fang Cheng, Olga Chertkov, Lynne Goodwin, Roxane Tapia, Cliff Han, Konstantinos Liolios, Natalia Ivanova, Konstantinos Mavromatis, Galina Ovchinnikova, Amrita Pati, Amy Chen, Krishna Palaniappan, Miriam Land, Loren Hauser, Yun-Juan Chang, Cynthia D. Jeffries, Rüdiger Pukall, Stefan Spring, Manfred Rohde, Johannes Sikorski, Markus Göker, Tanja Woyke, James Bristow, Jonathan A. Eisen, Victor Markowitz, Philip Hugenholtz, Nikos C. Kyrpides, Hans-Peter Klenk

**Affiliations:** 1 DOE Joint Genome Institute, Walnut Creek, California, USA; 2 HZI – Helmholtz Centre for Infection Research, Braunschweig, Germany; 3 Los Alamos National Laboratory, Bioscience Division, Los Alamos, New Mexico, USA; 4 Biological Data Management and Technology Center, Lawrence Berkeley National Laboratory, Berkeley, California, USA; 5 Oak Ridge National Laboratory, Oak Ridge, Tennessee, USA; 6 DSMZ - German Collection of Microorganisms and Cell Cultures GmbH, Braunschweig, Germany; 7 University of California Davis Genome Center, Davis, California, USA

**Keywords:** strictly anaerobic, obligate amino-acid-degrading, Gram-negative, nonmotile, asaccharolytic, mesophilic, chemoorganotrophic, *Synergistaceae*, *‘Synergistetes*’, GEBA

## Abstract

*Aminomonas paucivorans* Baena *et al.* 1999 is the type species of the genus *Aminomonas*, which belongs to the family *Synergistaceae*. The species is of interest because it is an asaccharolytic chemoorganotrophic bacterium which ferments quite a number of amino acids. This is the first finished genome sequence (with one gap in a rDNA region) of a member of the genus *Aminomonas* and the third sequence from the family *Synergistaceae*. The 2,630,120 bp long genome with its 2,433 protein-coding and 61 RNA genes is a part of the ***G****enomic* ***E****ncyclopedia of* ***B****acteria and* ***A****rchaea* project.

## Introduction

Strain GLU-3^T^ (= DSM 12260 = ATCC BAA-6) is the type strain of the species *Aminomonas paucivorans*, which in turn is the type and only species of the genus *Aminomonas* [1,2]. The generic name derives from the Latin word ‘aminum’ meaning ‘amine’ and the Greek word ‘*monas*’ meaning ‘a unit or monad’, referring to amine-degrading monads [2]. The species epithet is derived from the Latin word ‘*paucus*’ meaning ‘few or little’ and the Latin word ‘*vorans*’ meaning ‘digesting’, referring to digesting little [2]. Strain GLU-3^T^ was isolated from anaerobic sludge of a dairy wastewater treatment plant in SantaFe de Bogota, Colombia [2]. So far, no further isolates have been obtained for *A. paucivorans*. Here we present a summary classification and a set of features for *A. paucivorans* GLU-3^T^, together with the description of the non-contiguous finished genomic sequencing and annotation.

## Classification and features

**Figure 1:**
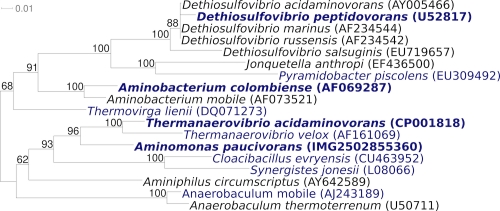
Phylogenetic tree highlighting the position of *A. paucivorans* GLU-3^T^ relative to the other type strains within the family *Synergistaceae*. The tree was inferred from 1,347 aligned characters [4,5] of the 16S rRNA gene sequence under the maximum likelihood criterion [6] and rooted in accordance with the current taxonomy. The branches are scaled in terms of the expected number of substitutions per site. Numbers above branches are support values from 1,000 bootstrap replicates [7] if larger than 60%. Lineages with type strain genome sequencing projects registered in GOLD [8] are shown in blue, published genomes in bold [3,9,10].

The 16S rRNA gene of *A. paucivorans* GLU-3^T^ shares 96% sequence identity with that of the type strain of *Thermanaerovibrio acidaminovorans*, which was isolated from an upflow anaerobic sludge bed reactor of a sugar refinery, Breda, the Netherlands  [3] (Figure 1), and 82.2-96.4% sequence identity with the type strains from the other members of the family *Synergistaceae* [11]. The sequences of four marine metagenomic clones in the env_nt database, 1096626071844 (AACY020063505), 1096626840052 (AACY020539193), 1096626748225 (AACY020105546) and 1096626774924 (AACY020274567) share 96% sequence identity with *A. paucivorans* GLU-3^T^ (as of October 2010). A representative genomic 16S rRNA sequence of *A. paucivorans* was compared using NCBI BLAST under default values with the most recent release of the Greengenes database [12] and the relative frequencies of taxa and keywords, weighted by BLAST scores, were determined. The four most frequent genera were *Thermanaerovibrio* (65.5%), *Aminomonas* (18.0%), *Anaerobaculum* (9.0%) and *Aminiphilus* (7.6%). The species yielding the highest score was *T. acidaminovorans*. The five most frequent keywords within the labels of environmental samples which yielded hits were 'anaerobic' (7.2%), 'sludge' (6.9%), 'wastewater' (6.8%), 'municipal' (6.8%) and 'digester' (6.7%). These keywords corroborate the physiological and ecological features on strain GLU-3^T^ as depicted in the original description [2].The single most frequent keyword within the labels of environmental samples which yielded hits of a higher score than the highest scoring species was 'harbor/sediment' (50.0%).

Figure 1 shows the phylogenetic neighborhood of *A. paucivorans* GLU-3^T^ in a 16S rRNA based tree. The sequences of the three 16S rRNA gene copies in the genome of *A. paucivorans* differ from each other by up to one nucleotide, and differ by up to eleven nucleotides from the previously published 16S rRNA sequence (AF072581), which contains 59 ambiguous base calls (ambiguous bases not count as differences).

**Figure 2:**
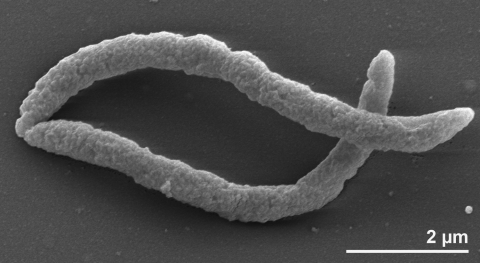
Scanning electron micrograph of *A. paucivorans* GLU-3^T^

*A. paucivorans* GLU-3^T^ is described as Gram-negative, slightly curved, rod-shaped bacterium (0.3 × 4.0-6.0 µm), which occurs singly or in pairs (Figure 2 and Table 1). Colonies of strain GLU-3^T^ are round, smooth and white, with a diameter up to 1 mm [2]. Strain GLU-3^T^ does not produce endospores [2]. The organism does not have flagella and motility is not observed [2], although plenty of motility genes are present in the genome. Strain GLU-3^T^ is a strictly anaerobic, mesophilic, chemoorganotrophic and asaccharolytic bacterium [2]. The temperature range for growth is 20-40°C, with an optimum at 35°C [2]. The pH range for growth is 6.7-8.3, with an optimum at 7.5 [2]. The organism does not require NaCl for growth but tolerates up to 2.0% [2]. The optimum growth occurs in media with 0.05-0.5% of NaCl [2]. The species requires yeast extract for growth [2]. The organism is able to ferment arginine, histidine, glutamine, threonine, and glycine [2]. Arginine is fermented to acetate, formate and ornithine [2]. Histidine is fermented to acetate and formate [2]. Glutamate is fermented to acetate, formate and trace amounts of propionate [2]. Threonine and glycine are fermented to acetate [2]. Casamino acid, peptone and cysteine are only poorly used by the strain GLU-3^T^, and acetate is the end-product of the amino acid metabolism [2]. A mixed culture of strain GLU-3^T^ and *Methanobacterium formicicum* does not extend the range of substrate utilization [2], as is observed for, e.g., *Aminobacterium colombiense* - [9]. Methane is not detectable in mixed cultures, when grown in glycine and threonine [2], however, the end-product profiles are the same as in pure culture [2]. The major end-product is shifted from acetate to propionate, when strain GLU-3^T^ was grown together with *M. formicicum* on arginine, histidine and glutamate [2]. Ornithine is not accumulated during arginine degradation in mixed culture [2]. Strain GLU-3^T^ does not degrade alanine and branched-chain amino acids, valine, leucine and isoleucine either in pure culture or in syntrophic growth with *M. formicicum* [2]. Also, the range of amino acid utilization is not increased in co-culture with *M. formicicum* [2]. Strain GLU-3^T^ does not grow on carbohydrates, gelatin, casein, pyruvate, succinate, malate, fumarate, *α*-ketoglutarate, mesaconate, *β*-methylaspartate, oxaloacetate, glycerol, ethanol, acetate, propionate, butyrate, lactate, citrate, leucine, lysine, alanine, valine, proline, serine, methionine, asparagines, phenylalanine and aspartate [2]. The organism does not utilize sulfate, thiosulfate, elemental sulfur, sulfite, nitrate and fumarate as electron acceptors [2].

**Table 1:** Classification and general features of *A. paucivorans* GLU-3^T^ according to the MIGS recommendations [13].

**MIGS ID**	**Property**	**Term**	**Evidence code**
	Current classification	Domain *Bacteria*	TAS [14]
		Phylum “*Synergistetes*”	TAS [15]
		Class *Synergistia*	TAS [15]
		Order *Synergistales*	TAS [15]
		Family *Synergistaceae*	TAS [15]
		Genus *Aminomonas*	TAS [2]
		Species *Aminomonas paucivorans*	TAS [2]
		Type strain GLU-3	TAS [2]
	Gram stain	negative	TAS [2]
	Cell shape	slightly curved rods occurring singly or in pairs	TAS [2]
	Motility	none	TAS [2]
	Sporulation	none	TAS [2]
	Temperature range	20°C-40°C	TAS [2]
	Optimum temperature	35°C	TAS [2]
	Salinity	0-2% NaCl (optimum 0.05-0.50%)	TAS [2]
MIGS-22	Oxygen requirement	strictly anaerobic	TAS [2]
	Carbon source	amino acids	TAS [2]
	Energy source	chemoorganotroph	TAS [2]
MIGS-6	Habitat	wastewater	TAS [2]
MIGS-15	Biotic relationship	free-living	NAS
MIGS-14	Pathogenicity	none	NAS
	Biosafety level	1	TAS [16]
	Isolation	anaerobic sludge of a dairy wastewater treatment plant	TAS [2]
MIGS-4	Geographic location	SantaFe de Bogota, Colombia	TAS [2]
MIGS-5	Sample collection time	1996	NAS
MIGS-4.1	Latitude	4.60	NAS
MIGS-4.2	Longitude	74.08	NAS
MIGS-4.3	Depth	not reported	
MIGS-4.4	Altitude	2620 m	NAS

Evidence codes - IDA: Inferred from Direct Assay (first time in publication); TAS: Traceable Author Statement (i.e., a direct report exists in the literature); NAS: Non-traceable Author Statement (i.e., not directly observed for the living, isolated sample, but based on a generally accepted property for the species, or anecdotal evidence). These evidence codes are from of the Gene Ontology project [17]. If the evidence code is IDA, then the property was directly observed by one of the authors or an expert mentioned in the acknowledgements.

### 

#### Chemotaxonomy

No chemotaxonomic data are currently available for *A. paucivorans* or for other members of the genus *Aminomonas*.

## Genome sequencing and annotation

### Genome project history

This organism was selected for sequencing on the basis of its phylogenetic position [18], and is part of the ***G****enomic* ***E****ncyclopedia of* ***B****acteria and* ***A****rchaea* project [19]. The genome project is deposited in the Genome OnLine Database [8,20] and the non-contiguous finished genome sequence has been deposited in DDBJ/EMBL/GenBank under the accession AEIV00000000. The version described in this paper is the first version, AEIV01000000. Sequencing, finishing and annotation were performed by the DOE Joint Genome Institute (JGI). A summary of the project information is shown in Table 2.

**Table 2:** Genome sequencing project information

**MIGS ID**	**Property**	**Term**
MIGS-31	Finishing quality	Non-contiguous finished
MIGS-28	Libraries used	Three genomic libraries: one 454 pyrosequence standard library, 454 PE library (12 kb insert size), one Illumina standard library
MIGS-29	Sequencing platforms	454 GS FLX Titanium, Illumina GAii
MIGS-31.2	Sequencing coverage	202.0 × Illumina; 72.4× pyrosequence
MIGS-30	Assemblers	Newbler version 2.0.00.20- PostRelease-11-05-2008-gcc-3.4.6, phrap
MIGS-32	Gene calling method	Prodigal 1.4, GenePRIMP
	INSDC ID	CM001022, AEIV00000000
	Genbank Date of Release	November 2, 2010
	GOLD ID	Gi02542
	NCBI project ID	33371
	Database: IMG-GEBA	2502790015
MIGS-13	Source material identifier	DSM 12260
	Project relevance	Tree of Life, GEBA

### Growth conditions and DNA isolation

*A. paucivorans* GLU-3^T^, DSM 12260, was grown anaerobically in DSMZ medium 846 (Anaerobic Serine/Arginine medium) [21] at 37°C. DNA was isolated from 0.5-1 g of cell paste using the MasterPure Gram-positive DNA purification kit (Epicentre MGP04100) following the standard protocol as recommended by the manufacturer, with modification st/LALM for cell lysis as described in Wu *et al.* [19].

### Genome sequencing and assembly

The genome was sequenced using a combination of Illumina and 454 sequencing platforms. All general aspects of library construction and sequencing can be found at the JGI website [22]. Pyrosequencing reads were assembled using the Newbler assembler version 2.0.00.20-PostRelease-11-05-2008-gcc-3.4.6 (Roche). The initial Newbler assembly consisted of 126 contigs in 103 scaffolds and was converted into a phrap assembly by making fake reads from the consensus for collecting the read pairs in the 454 paired end library. Illumina GAii sequencing data (525.3 Mb) was assembled with Velvet [23] and the consensus sequences were shredded into 1.5 kb overlapped fake reads and assembled together with the 454 data. The 454 draft assembly was based on 190.7 Mb 454 draft data and all of the 454 paired end data. Newbler parameters were -consed -a 50 -l 350 -g -m -ml 20.

The Phred/Phrap/Consed software package [24] was used for sequence assembly and quality assessment in the subsequent finishing process. After the shotgun stage, reads were assembled with parallel phrap (High Performance Software, LLC). Possible mis-assemblies were corrected with gapResolution (http://www.jgi.doe.gov/), Dupfinisher, or sequencing cloned bridging PCR fragments with subcloning or transposon bombing (Epicentre Biotechnologies, Madison, WI) [25]. Gaps between contigs were closed by editing in Consed, by PCR and by Bubble PCR primer walks (J.-F.Chang, unpublished). A total of 259 additional reactions were necessary to close gaps and to raise the quality of the finished sequence. Illumina reads were also used to correct potential base errors and increase consensus quality using a software (Polisher) developed at JGI [26]. The error rate of the completed genome sequence is less than 1 in 100,000. Together, the combination of the Illumina and 454 sequencing platforms provided 274.4× coverage of the genome. The final assembly contained 535, 052 pyrosequences and 15,007,632 Illumina reads.

### Genome annotation

Genes were identified using Prodigal [27] as part of the Oak Ridge National Laboratory genome annotation pipeline, followed by a round of manual curation using the JGI GenePRIMP pipeline [28]. The predicted CDSs were translated and used to search the National Center for Biotechnology Information (NCBI) nonredundant database, UniProt, TIGR-Fam, Pfam, PRIAM, KEGG, COG, and InterPro databases. Additional gene prediction analysis and functional annotation was performed within the Integrated Microbial Genomes - Expert Review (IMG-ER) platform [29].

## Genome properties

**Figure 3:**
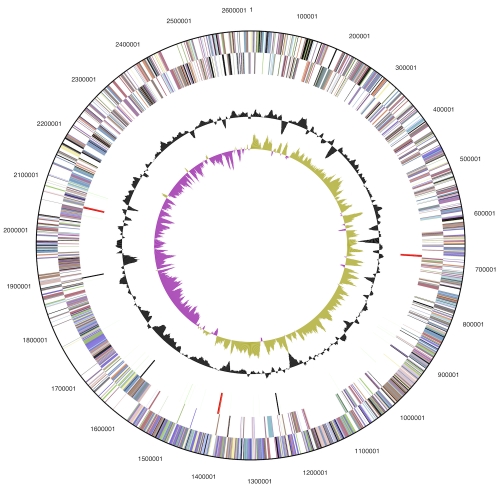
Graphical circular map of the genome. From outside to the center: Genes on forward strand (color by COG categories), Genes on reverse strand (color by COG categories), RNA genes (tRNAs green, rRNAs red, other RNAs black), GC content, GC skew.

The genome consists of a 2,630,120 bp long chromosome with an overall GC content of 67.6% (Table 3 and Figure 3). Of the 2,494 genes predicted, 2,433 were protein-coding genes, and 61 RNAs; 34 pseudogenes were also identified. The majority of the protein-coding genes (77.2%) were assigned with a putative function while the remaining ones were annotated as hypothetical proteins. The distribution of genes into COGs functional categories is presented in Table 4.

**Table 3:** Genome Statistics

**Attribute**	**Value**	**% of Total**
Genome size (bp)	2,630,120	100.00%
DNA Coding region (bp)	2,411,389	91.68%
DNA G+C content (bp)	1,777,554	67.59%
Number of replicons	1	
Extrachromosomal elements	0	
Total genes	2,494	100.00%
RNA genes	61	2.45%
rRNA operons	3	
Protein-coding genes	2,433	97.55%
Pseudo genes	34	1.36%
Genes with function prediction	1,926	77.23%
Genes in paralog clusters	338	13.55%
Genes assigned to COGs	1,988	79.71%
Genes assigned Pfam domains	2,047	82.08%
Genes with signal peptides	446	17.88%
Genes with transmembrane helices	588	23.58%
CRISPR repeats	4	

**Table 4:** Number of genes associated with the general COG functional categories

**Code**	**value**	**%age**	**Description**
J	158	7.2	Translation, ribosomal structure and biogenesis
A	0	0.0	RNA processing and modification
K	138	6.3	Transcription
L	107	4.9	Replication, recombination and repair
B	0	0.0	Chromatin structure and dynamics
D	29	1.3	Cell cycle control, cell division, chromosome partitioning
Y	0	0.0	Nuclear structure
V	33	1.5	Defense mechanisms
T	154	7.0	Signal transduction mechanisms
M	123	5.6	Cell wall/membrane/envelope biogenesis
N	90	4.1	Cell motility
Z	0	0.0	Cytoskeleton
W	0	0.0	Extracellular structures
U	48	2.2	Intracellular trafficking, secretion, and vesicular transport
O	64	2.9	Posttranslational modification, protein turnover, chaperones
C	161	7.3	Energy production and conversion
G	104	4.7	Carbohydrate transport and metabolism
E	251	11.4	Amino acid transport and metabolism
F	71	3.2	Nucleotide transport and metabolism
H	111	5.0	Coenzyme transport and metabolism
I	35	1.6	Lipid transport and metabolism
P	102	4.6	Inorganic ion transport and metabolism
Q	25	1.1	Secondary metabolites biosynthesis, transport and catabolism
R	236	10.7	General function prediction only
S	166	7.5	Function unknown
-	506	20.3	Not in COGs
